# Specialized prefrontal “auditory fields”: organization of primate prefrontal-temporal pathways

**DOI:** 10.3389/fnins.2014.00077

**Published:** 2014-04-16

**Authors:** Maria Medalla, Helen Barbas

**Affiliations:** ^1^Department of Anatomy and Neurobiology, Boston UniversityBoston, MA, USA; ^2^Neural Systems Laboratory, Department of Health Sciences, Boston UniversityBoston, MA, USA; ^3^Department of Health Sciences, Boston UniversityBoston, MA, USA

**Keywords:** frontopolar cortex, frontal pole, area 10, anterior cingulate cortex, synaptic pathways, inhibitory neurons, laminar connections

## Abstract

No other modality is more frequently represented in the prefrontal cortex than the auditory, but the role of auditory information in prefrontal functions is not well understood. Pathways from auditory association cortices reach distinct sites in the lateral, orbital, and medial surfaces of the prefrontal cortex in rhesus monkeys. Among prefrontal areas, frontopolar area 10 has the densest interconnections with auditory association areas, spanning a large antero-posterior extent of the superior temporal gyrus from the temporal pole to auditory parabelt and belt regions. Moreover, auditory pathways make up the largest component of the extrinsic connections of area 10, suggesting a special relationship with the auditory modality. Here we review anatomic evidence showing that frontopolar area 10 is indeed the main frontal “auditory field” as the major recipient of auditory input in the frontal lobe and chief source of output to auditory cortices. Area 10 is thought to be the functional node for the most complex cognitive tasks of multitasking and keeping track of information for future decisions. These patterns suggest that the auditory association links of area 10 are critical for complex cognition. The first part of this review focuses on the organization of prefrontal-auditory pathways at the level of the system and the synapse, with a particular emphasis on area 10. Then we explore ideas on how the elusive role of area 10 in complex cognition may be related to the specialized relationship with auditory association cortices.

## Overview

It is quite remarkable that there is not a waking moment that is completely free of sound. Whether it is the buzzing of our surroundings or on-going conversations, our minds are bombarded by endless streams of auditory signals [e.g., (Conway et al., 2001; Denham and Winkler, 2006; Jaaskelainen et al., 2007; Micheyl et al., 2007); reviewed in (Bee and Micheyl, 2008; Winkler et al., 2009)]. Superimposed on the external auditory environment is an inward stream of thoughts akin to the external that contributes to the sea of auditory signals [e.g., (Scott et al., 2013b); reviewed in (Haykin and Chen, 2005; Allen et al., 2008; Perrone-Bertolotti et al., 2014)]. But what is more remarkable is our ability to sort out what is important in this sea of noise. The prefrontal cortex is necessary for the function of selecting relevant information and suppressing irrelevant signals for the task at hand (reviewed in Knight et al., 1999; Miller and Cohen, 2001). The interaction of prefrontal cortices with auditory association cortices provides an excellent demonstration of this prefrontal executive function (Chao and Knight, 1997), which is thought to reach beyond auditory processing *per se*, and extend to the global process of “using our thoughts” to guide cognitive tasks [e.g., (Frith, 1996; Wenzlaff and Wegner, 2000; Brewin and Smart, 2005); reviewed in (Knight et al., 1999; Allen et al., 2008; Winkler et al., 2009; Perrone-Bertolotti et al., 2014)]. The behavioral exemplars of these prefrontal-auditory interactions are evident in our daily lives—from following a conversation in a crowded room or tackling an inner debate on what to order from a menu—but the neural substrate and mechanisms are unclear.

**Figure 1:**
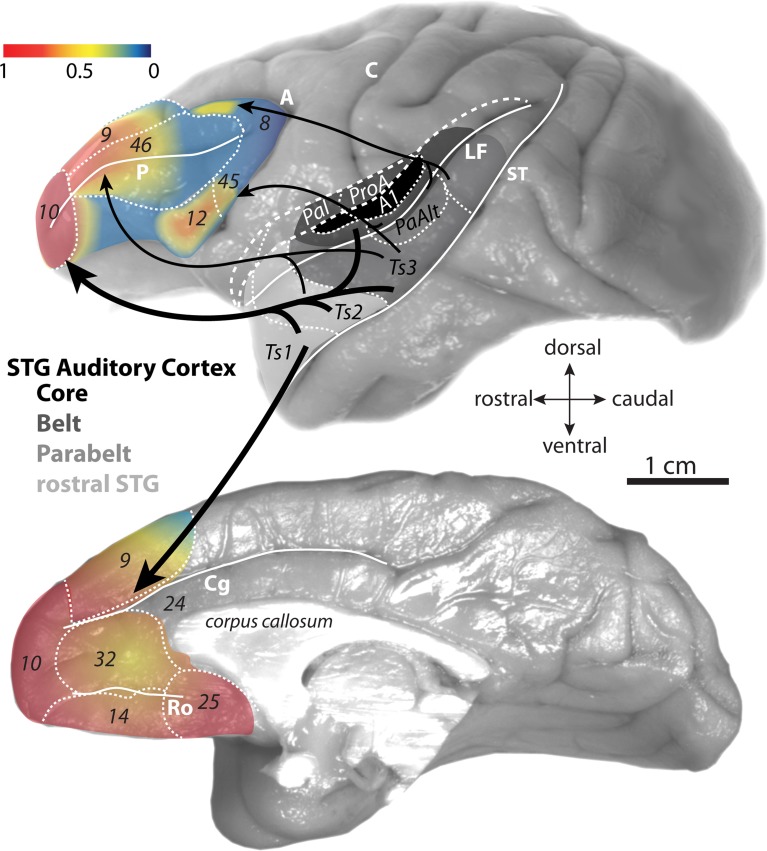
**Gradient map of auditory input to the prefrontal cortex.** Lateral (top) and medial (bottom) surfaces of the rhesus monkey brain show proportions of afferent pathways (projection neurons) from STG to prefrontal cortex. The proportions (derived from Barbas and Mesulam, 1985 and Barbas et al., 1999) are normalized against the highest number in the set and expressed as a gradient map. The relative proportions are also represented by the weights of the arrows denoting the STG→prefrontal pathways. Architectonic areas of the rhesus monkey auditory cortices in the STG are according to map of Galaburda and Pandya (1983) and functional divisions are according to Hackett et al. (1999). Long dashes demarcate banks of sulci schematically unfolded and short dashes delineate areal boundaries. Abbreviations for sulci: A, arcuate; C, central; Cg, cingulate; LF, lateral fissure; Ro, rostral; ST, superior temporal.

From a neuroanatomical perspective, the importance of auditory information in prefrontal function is intuitive given that no other sensory modality is more frequently and vastly represented in the prefrontal cortex than the auditory modality (for review see Barbas et al., 2002). Pathways from auditory association cortices reach lateral, medial, and orbital surfaces of the prefrontal cortex. But the densest prefrontal interconnections with auditory association areas are with the frontopolar cortex, area 10, which mediates the most complex and abstract cognitive tasks (Figure 1; reviewed in Barbas et al., 2002; Burgess et al., 2007; Koechlin and Hyafil, 2007; Smith et al., 2007; Badre and D'Esposito, 2009).

The frontopolar cortex is situated in the most anterior part of the prefrontal cortex, extending from the lateral to the medial and orbital surfaces (Barbas and Pandya, 1989; Petrides, 2005). The function of this region had remained elusive and it was not until the advent of human functional neuroimaging that its role in complex cognition began to emerge (reviewed in Burgess et al., 2007; Koechlin and Hyafil, 2007). Early detailed physiologic work on prefrontal function in non-human primates had focused on visual-related processing, specifically the caudal periarcuate area 46 and area 8, the frontal eye fields (FEF) (Jacobsen, 1936; Robinson and Fuchs, 1969; Fuster, 1973; Niki and Watanabe, 1976). Studies in these periarcuate regions have led to important findings on the role of the prefrontal cortex in the active maintenance of information for a task at hand (working memory) and attention (reviewed in Goldman-Rakic, 1995; Fuster, 2001; Constantinidis and Procyk, 2004; Funahashi, 2006). While these findings are pivotal to our understanding of the prefrontal cortex, it is often not emphasized that visual information in lateral prefrontal cortex is represented in rather restricted areas within the caudal periarcuate region and at the most posterior part of the principal sulcus (Barbas and Mesulam, 1981, 1985; Barbas, 1988; Bullier et al., 1996).

**Figure 2:**
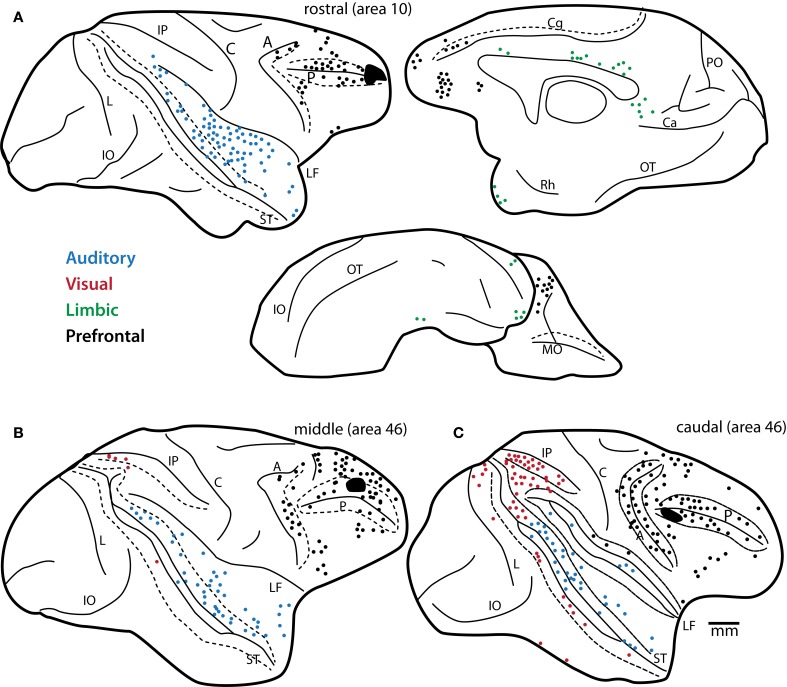
**Predominance of auditory projection neurons directed to area 10.** Distribution of projection neurons from STG to area 10 in comparison to lateral prefrontal areas. Maps show plots of projection neurons after injection of retrograde neural tracers in **(A)** rostral (area 10 injection), **(B)** middle (mid-area 46, dorsal) and **(C)** caudal (caudal area 46) areas of the lateral prefrontal cortex. Note the decrease in auditory projection neurons (blue) and increase in visual projection neurons (red) in the caudal site **(C)**, shown on the lateral surface. In **(A)** a complete map of pathways directed to area 10 shows projection neurons in prefrontal (black dots), auditory (blue), and limbic (green) cortices on the lateral, medial and orbital surfaces of the rhesus monkey brain. Note the predominance of auditory projection neurons directed to area 10 from extrinsic sources outside the prefrontal cortex. Long dashes demarcate banks of sulci schematically unfolded. Abbreviations for sulci: A, arcuate; C, central; Ca, calcarine; Cg, cingulate; IO, inferior occipital; IP, intraparietal; L, lunate; LF, lateral fissure; MO, medial orbital; OT, occipitotemporal; PO, parieto-occipital; Rh, rhinal; ST, superior temporal. Adapted from Barbas and Mesulam, 1985.

Early anatomic studies showed that visual and visuomotor connections were confined to caudal lateral prefrontal areas (Barbas and Mesulam, 1981, 1985). Moreover, the studies revealed a striking opposite gradient in visual and auditory input along the rostro-caudal extent of the principal sulcus in rhesus monkeys (Barbas and Mesulam, 1985). While visual input was robust caudally, projections from auditory association cortices were sparse in caudal peri-principalis regions and became progressively denser in rostral areas, with the densest auditory pathways directed to the rostral frontopolar cortex in area 10 (Figure 2). While classical and modern anatomic studies have found strong auditory-related connections in the frontal pole (Pandya and Kuypers, 1969; Chavis and Pandya, 1976; Petrides and Pandya, 1988; Barbas et al., 1999, 2005; Hackett et al., 1999; Romanski et al., 1999a,b; Medalla et al., 2007), it is generally understated in the functional literature. This is in striking contrast to the well-known functional involvement of the caudal periarcuate FEF in visual tasks [e.g., (Mishkin, 1966; Robinson and Fuchs, 1969; Mohler et al., 1973); reviewed in (Bruce and Goldberg, 1984; Schiller and Tehovnik, 2001; Schall and Boucher, 2007)]. The extrinsic connections of the frontal pole are nearly exclusively with auditory association areas in rhesus monkeys, which rival in strength the visual-related connections of the FEF [(Barbas and Mesulam, 1985); reviewed in (Barbas et al., 2002; Lynch and Tian, 2005)]. Moreover, while most of the prefrontal cortex receives highly-processed high-order sensory information, both area 10 and the FEF receive projections from relatively “early” unimodal sensory association cortices as well [(e.g., auditory belt and parabelt areas for area 10, and areas V2 and V4 for FEF); (Barbas and Mesulam, 1985); reviewed in (Barbas et al., 2002)]. Early-processing sensory areas receive strong direct input from the primary cortices [e.g., (Pandya et al., 1988; Rauschecker et al., 1997; Kaas and Hackett, 1998)]. In this light, the frontopolar area 10 may be regarded as the frontal “auditory field,” to reflect the emphasis of its rich and varied bidirectional connections with auditory association cortices.

## Organization of auditory-related prefrontal areas

While auditory connections predominate for area 10 among prefrontal areas, auditory input impinges on several prefrontal auditory “hotspots” on the lateral and medial surfaces (Figure 1; Pandya and Kuypers, 1969; Chavis and Pandya, 1976; Barbas and Mesulam, 1981, 1985; Petrides and Pandya, 1988, 2002; Morel et al., 1993; Kaas and Hackett, 1998; Barbas et al., 1999, 2005; Hackett et al., 1999; Romanski et al., 1999a,b; Medalla et al., 2007). Auditory pathways also reach areas within the largely multimodal orbitofrontal cortex, but this is discussed elsewhere (reviewed in Barbas et al., 2011).

### Auditory association cortices that are linked with prefrontal cortices

The auditory cortices that are most strongly connected with prefrontal areas lie within the superior temporal gyrus (STG), extending from the inferior bank of the lateral fissure to the upper (medial) bank of the superior temporal sulcus (Figure 1, top). This temporal region is subdivided into distinct areas, according to the maps of Galaburda and Pandya (1983) and Hackett et al. (1999). These areas fall within four main subdivisions of the functional map of the auditory cortex: the core area, which includes the primary auditory cortex; the adjacent belt and parabelt region, and the anterior temporal polar region (reviewed in Romanski and Averbeck, 2009; Figure 1, top).

The prefrontal cortex is interconnected roughly with the anterior two thirds of STG, which extends from temporal polar cortex through anterior parabelt and belt areas (Figure 1, top). These parts of STG consist mostly of high-order auditory association areas that respond to complex auditory stimuli [(Plakke et al., 2013; Ng et al., 2014); reviewed in (Romanski and Averbeck, 2009)]. A small subset of prefrontal interconnections include caudal auditory belt areas (Figure 1, top; Hackett et al., 1999; Romanski et al., 1999a,b). Physiologic and metabolic mapping studies show activation of these temporal and interconnected prefrontal areas in response to auditory stimuli (Rauschecker et al., 1997; Poremba et al., 2004; Plakke et al., 2013; Ng et al., 2014). Details of the organization of the auditory cortex can be found elsewhere (reviewed in Romanski and Averbeck, 2009). This review focuses specifically on the anatomic organization of prefrontal-temporal pathways that may shed light on the mechanism of communication and information transfer within a network for high-order cognition.

### Topography of prefrontal “hotspots” for auditory inputs and outputs

In the lateral prefrontal cortex, there is a graded increase in the density of auditory connections along the caudal to rostral axis (Figure 2; Barbas and Mesulam, 1985). Within the caudal lateral prefrontal cortex, auditory input is relatively restricted to specific domains of rostral dorsal area 8 (Barbas and Mesulam, 1981) and areas 45 and 12 in the ventrolateral prefrontal cortex (Figure 1, top; Hackett et al., 1999; Romanski et al., 1999a,b). These areas receive pathways from auditory association cortices from a restricted and more caudal part of STG, within the parabelt and belt areas (Figure 1, top; Barbas and Mesulam, 1981, 1985; Hackett et al., 1999; Romanski et al., 1999a,b). These areas also receive significant projections from visual association cortices and are thought to be sites of visual-auditory convergence in the prefrontal cortex [(Barbas, 1988); reviewed in (Romanski, 2007)]. Electrical stimulation of rostral dorsal area 8 (area 8a) in the upper bank of the arcuate sulcus, elicits large amplitude saccades (Robinson and Fuchs, 1969; Mohler et al., 1973; Bruce and Goldberg, 1985) and has been discussed as a region that may help direct attention to peripheral visual and auditory stimuli [e.g., (Barbas and Mesulam, 1981)]. Ventrolateral areas 12 and 45 have been the most well studied in terms of single-unit recordings during auditory tasks in awake behaving monkeys. In the ventrolateral prefrontal cortex, neurons are responsive to complex acoustic stimuli, including species-specific vocalizations that involve a complex interplay of visual and auditory information (reviewed in Romanski and Averbeck, 2009). In the more anterior lateral prefrontal areas, mid-dorsolateral areas 46 and 9, pathways from auditory cortices are stronger as visual input wanes (Figures 2B,C). This mid-dorsolateral prefrontal cortex is thought to play a prominent role in classic working memory-temporary active maintenance of information needed to perform the task at hand (reviewed in Levy and Goldman-Rakic, 2000; Petrides, 2005).

Unlike the restricted patches in lateral prefrontal cortices that have connections with auditory association cortices, the representation of the auditory modality is widespread in the medial prefrontal cortex (Figure 1). Dorsolateral area 9 extends to the medial surface, where there is graded increase in connections with auditory cortices (Barbas et al., 1999). Dense auditory pathways along the medial wall reach all the way from rostral medial prefrontal area 10 to more caudally situated areas 14, 32, and 25 (Figure 1, bottom; Barbas et al., 1999). In particular, auditory connections in areas 32 and 25 of the anterior cingulate cortex (ACC) rival in density auditory connections with area 10. However, unlike area 10, which is privy to information from both early and high-order auditory association cortices, the ACC has access only to highly-processed auditory information through dense interconnections with the rostral STG, especially with areas near the temporal pole [(Barbas et al., 1999); reviewed in (Barbas et al., 2002)]. Nonetheless, with its robust anatomic links with the rostral auditory association cortices, the ACC can be regarded as the medial frontal auditory “hotspot.” In fact, the ACC has a demonstrated robust and functional interaction with auditory areas. Electrical stimulation of the ACC can evoke species-specific vocalizations in monkeys, a pathway thought to mediate emotional communication [(Muller-Preuss et al., 1980; Muller-Preuss and Ploog, 1981); reviewed in (Vogt and Barbas, 1988)]. Activity in the ACC has also been correlated with auditory processing of actual and internal speech in humans (Frith et al., 1995; McGuire et al., 1995).

In contrast to the lateral and medial prefrontal cortices described above, the role of auditory connections in area 10 processing is largely unknown. It was not until recently that the first electrophysiologic recordings from individual neurons in area 10 of non-human primates were conducted, ironically using visual stimuli (Tsujimoto et al., 2010). In that study, a subset of neurons in area 10 showed decision-selective activity but only during the feedback period of a visual working memory task. In humans, highly complex cognitive tasks that engage area 10 also entail high-order verbal processing [e.g., (Brewin and Smart, 2005; Bunge et al., 2005; Christoff et al., 2011); reviewed in (Wenzlaff and Wegner, 2000; Burgess et al., 2007; Koechlin and Hyafil, 2007; Badre and D'Esposito, 2009)]. Nonetheless, the functionally unexplored frontopolar-auditory network is anatomically robust and in recent years we have used high-resolution tract-tracing and imaging techniques to elucidate the organization of these pathways at the level of the system and the synapse.

## Structural organization of frontopolar area 10

**Figure 3:**
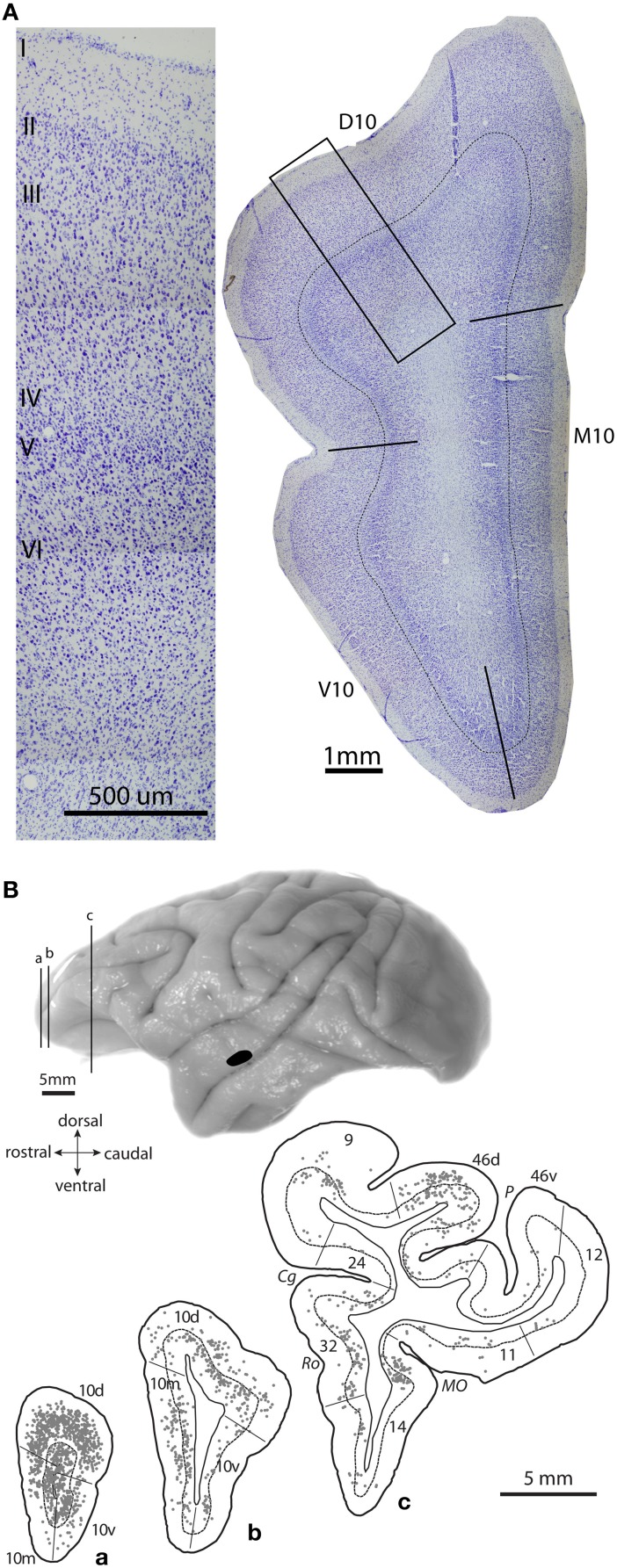
**Architecture of area 10 and prefrontal projection neurons to auditory cortex. (A)** Photomicrograph of a coronal Nissl-stained section shows the cytoarchitecture of area 10 in a rhesus monkey brain, with delineated dorsal, medial and ventral subregions (right). Thin line marks the top of layer IV. Inset shows location of a higher magnification photomicrograph of dorsal area 10 (left). **(B)** Lateral view of a rhesus monkey brain shows injection site of a retrograde tracer in STG area Ts2 (black); **(a–c)**, Coronal sections show plots of projection neurons in prefrontal cortices directed to the STG site in area Ts2. Adapted from Medalla et al., 2007.

Frontopolar area 10 in rhesus monkeys is a granular cortical area with a well-defined layer IV; it encompasses about the anterior quarter of the prefrontal cortex (Figure 3A; Barbas and Pandya, 1989). Area 10 has expanded significantly in humans, especially on the lateral surface, concomitant with the expansion of other lateral prefrontal areas (Semendeferi et al., 2011). Recent mapping studies that compare functional coupling of activation across cortical areas in humans and monkeys have shown that the entire macaque area 10 corresponds only to the medial part of the human frontal pole (Sallet et al., 2013; Neubert et al., 2014).

**Figure 4:**
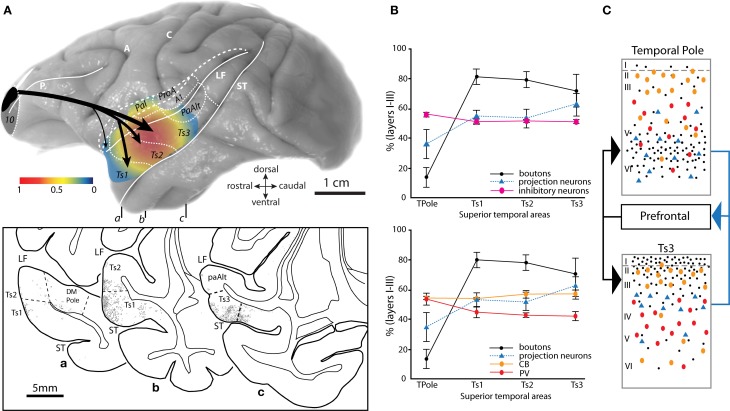
**Topography and laminar terminals of pathways from area 10 in distinct auditory cortices. (A)** Gradient map shows the relative density of area 10 pathway terminations in distinct STG areas. Axon terminals were labeled after injection of anterograde tracers in dorsal area 10. Density is normalized to the highest in the set. Long dashes demarcate banks of sulci schematically unfolded; short dashes delineate areal boundaries. Bottom inset shows coronal sections **(a–c)** through STG with plots of labeled terminations from area 10. Rostro-caudal level of each section is indicated on the whole brain (top). **(B)** The laminar distribution (expressed as percent in the upper layers I–III) of prefrontal interconnections relative to the laminar distribution of inhibitory neurons labeled with PV and CB in distinct STG areas. Top panel shows the combined proportion of CB and PV, bottom panel shows CB and PV proportions depicted separately. **(C)** Schematic summarizes the predominant pattern of connections (boutons, black dots; projection neurons, blue triangles) of prefrontal cortices with the agranular and dysgranular (limbic) parts of the temporal pole (top) and with a caudal eulaminate area of the superior temporal cortex (bottom), and their relationship to PV+ (red ovals) and CB+ (orange ovals) inhibitory interneurons. Black arrows (left) show the predominant laminar termination of prefrontal axons in superior temporal areas; blue arrow (right) shows the predominant laminar origin of projection neurons in superior temporal areas directed to prefrontal cortex. Abbreviations as in Figures 1, 2. Adapted from Barbas et al., 2005.

In the rhesus monkey, area 10 has dorsal, medial and ventral (basal) subdivisions, all of which are interconnected with auditory areas of the STG (Figures 3A, right, **3B**; Pandya and Kuypers, 1969; Chavis and Pandya, 1976; Petrides and Pandya, 1988). In addition to auditory connections, area 10 is heavily interconnected with other parts of the prefrontal cortex, especially dorsolateral prefrontal areas 9/46 and ACC area 32 [Figure 2A, black; (Barbas and Pandya, 1989; Barbas et al., 1999; Medalla and Barbas, 2010); reviewed in (Barbas et al., 2002)]. With regard to the extrinsic connections of area 10 outside the prefrontal cortex, most are with auditory association areas (Figure 2A, blue), and the rest are with the cortical limbic system in the cingulate, retrosplenial, rhinal, and anterior temporal polar cortices [Figure 2A, green; (Barbas and Mesulam, 1985; Barbas et al., 1999); reviewed in (Barbas et al., 2002)]. Area 10 receives massive input from a wide spectrum of STG areas, spanning from rostral sectors (temporal pole, Ts1-2 or STGr) to the more caudally situated anterior parabelt (area Ts3 or areas RP/RTL/AL) and belt (PaI/RTM and ProA/RM) auditory association cortices (Figures 1A, 2A; Barbas and Mesulam, 1981, 1985; Hackett et al., 1999; Romanski et al., 1999a,b). The reciprocal connections from area 10 to STG are also robust, encompassing a similarly widespread rostro-caudal extent along the STG (Figures 3B, 4A; Barbas et al., 2005; Germuska et al., 2006; Medalla et al., 2007). In recent years, we have investigated the fine structural features of these pathways from area 10 to STG to shed light on the potential influence of frontopolar area 10 on auditory processing within the STG.

## Frontopolar area 10 pathways to auditory association cortices

### Graded laminar terminations from area 10 to distinct auditory association areas

Pathways from area 10 terminate densely in rostral parts of STG and extend caudally to auditory parabelt and belt areas (Figure 4A; Barbas et al., 2005; Germuska et al., 2006; Medalla et al., 2007). The highest densities of axon terminals from area 10 reach areas Ts1-3, especially anterior parabelt area Ts3 (Figure 4A). There is a graded pattern of terminations in the cortical layers targeted by area 10 in STG (Figures 4B,C). Interestingly, this graded pattern of laminar termination coincides with graded changes in cortical structure of the targeted cortices in STG, characterized by an increase in neuronal density and granularity (appearance of layer IV) from temporal polar areas to more posterior STG cortices (Barbas and Rempel-Clower, 1997; Rempel-Clower and Barbas, 2000; Barbas et al., 2005). A large part of the temporal polar cortex is limbic type of cortex, characterized by an absent layer IV (agranular) or a poorly delineated layer IV (dysgranular). Posterior association areas are granular, with well-delineated cortical layers (Galaburda and Pandya, 1983). Axon terminals from area 10 are densest in the deep cortical layers (V–VI) of the agranular/dysgranular STG cortices in the temporal pole (Figures 4B, black dots; **4C** top), but are densest in the upper layers (I–IIIa) of the granular posterior auditory association areas Ts1-3 (Figures 4B, black dots, **4C** bottom; Barbas et al., 2005). This pattern is consistent with our structural model for cortico-cortical connections, which relates the laminar pattern of connections to the structural difference between interconnected areas (Barbas and Rempel-Clower, 1997; Rempel-Clower and Barbas, 2000; Barbas et al., 2005; Medalla and Barbas, 2006). Pathways that terminate in the upper layers emanate from areas that have a simpler laminar structure than the area of termination (e.g., a pathway from an agranular to a granular cortex). Pathways that terminate in the middle-deep layers link areas with the opposite structural relationship.

The significance of laminar termination patterns is that cortical layers are also distinct in terms of the excitatory and inhibitory neuronal microenvironment in different auditory association cortices [Figures 4B,C; (Barbas et al., 2005); reviewed in (Barbas et al., 2002)]. In the temporal pole, fibers from area 10 are concentrated in the middle-deep layers where they overlap with the dense population of excitatory layer V–VI pyramidal neurons that project back to area 10, but largely avoid the large population of inhibitory neurons found in the upper layers (Figures 4B,C, top). In contrast, axon fibers from area 10 reach primarily the upper layers of auditory association areas Ts1-3, where they co-mingle with the population of excitatory projection neurons in layers II–III that project to area 10, as well as the population of calbindin and calretinin inhibitory neurons, which are dense in the upper layers (Figures 4B,C, bottom). Thus, while there is a laminar “match” with regard to the prevalence of excitatory connections and inhibitory neurons in the auditory association areas Ts1-3, there is a laminar “mismatch” in the temporal pole where excitatory connections predominate in the deep layers but most inhibitory neurons are found in the upper layers in this region (Barbas et al., 2005).

### Laminar specific synaptic features of area 10 terminations in auditory association cortex

**Figure 5:**
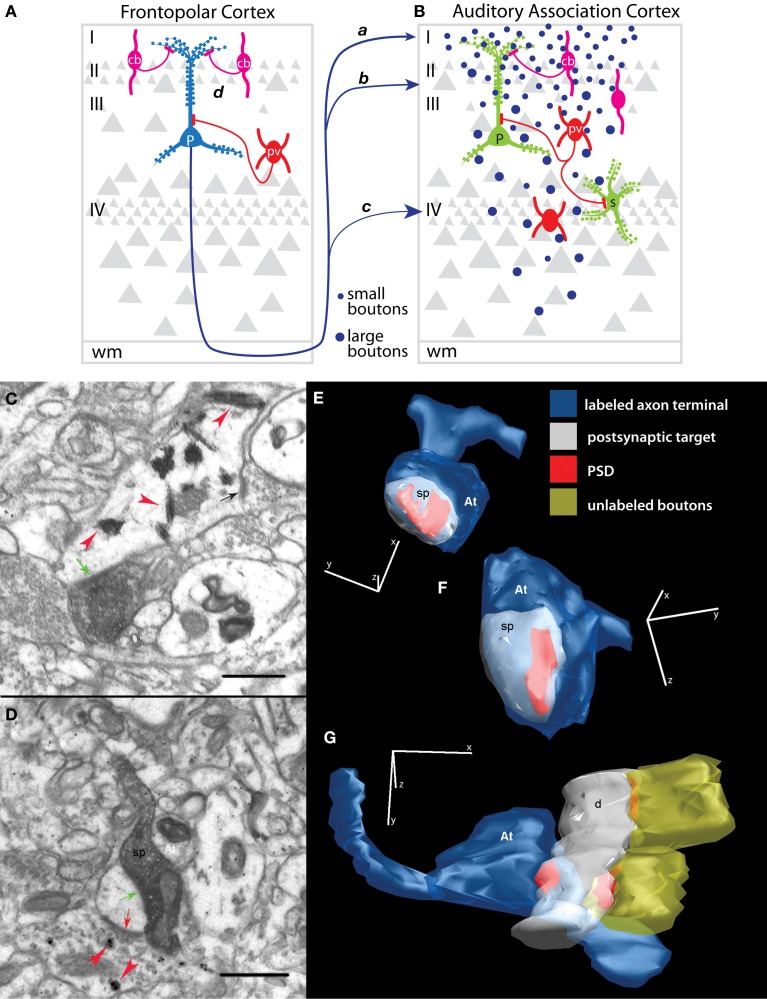
**Synapses of prefrontal axons in auditory association cortex. (A,B)** Schematic summarizes the predominant synaptic connections of the pathway from frontopolar area 10 to auditory association areas Ts1-3. The pathway from area 10, which terminates predominantly in the upper layers, shows a progressive increase in the volume of axon terminals **(B**, blue dots**)** from smallest in layer I (pathway *a*), through layers II–IIIa (*b*) and largest in layer IV (*c*). Boutons that terminate in different layers interact with specific excitatory (green) and inhibitory (red and magenta) dendritic domains and possibly with distinct populations of inhibitory neurons. Area 10 innervates mostly spines of pyramidal neurons (P) in layers I–IIIa, but may interact with other excitatory neuronal types, such as the spiny stellate neurons (s) in layer IV. Among the minority of area 10 axons that innervate inhibitory neurons, synapses are formed on both PV+ (red) and CB+ (magenta) inhibitory neurons that inhibit specific dendritic domains of pyramidal neurons. Inhibitory control may also occur at the site of origin of the pathway **(A**, pathway *d***)** through inhibition of STG-directed projection neurons in area 10 (P, blue). STG-directed projection neurons are dense in the upper layers of area 10, with apical dendrites overlapping extensively with CB+ inhibitory neurons. **(C)** Example of an electron micrograph shows tracer-labeled bouton from prefrontal cortex forming an asymmetric (excitatory) synapse (green arrow) on a PV+ dendrite (red arrowheads) in STG. Note the nearby unlabeled synapse (black arrow) on the PV+ dendritic shaft. **(D)** An electron micrograph shows a labeled prefrontal bouton forming a synapse with a spine in STG (green arrow). Note that the spine receives a symmetric (inhibitory) synapse (red arrow) from a CB+ inhibitory terminal (red arrowheads). **(E–G)** Examples of three-dimensional reconstructions from serial sections through labeled presynaptic axon terminals (At, blue) from prefrontal pathways and their corresponding postsynaptic densities (PSD, red) and postsynaptic targets in STG photographed in the electron microscope. **(E)** A small and **(F)** a large prefrontal bouton (At) each form a synapse (PSD) on a spine (sp, white). **(G)** A prefrontal bouton forms a synapse with a smooth/aspiny dendrite from a presumed inhibitory neuron in STG. Note the lack of spines and presence of nearby synapses on the shaft from unlabeled boutons, characteristics of smooth dendrites of inhibitory neurons. Adapted from Medalla et al., 2007.

Our recent work has focused on the pathways within the prefrontal-auditory network at the synaptic level, including features of axon terminals from area 10 to distinct cortical layers of STG areas Ts1-2 (Figures 5A,B, pathways *a, b, c*). We found a progressive increase in the size (volume) of area 10 axon terminals (boutons) in STG from layer I (Figures 5A,B, *a*), to layers II–IIIa (Figures 5A,B, *b*), and to the middle layer IV (Figures 5A,B, *c*; Germuska et al., 2006; Medalla et al., 2007). Thus, boutons from area 10 (Figure 5B, blue dots) in layer IV are larger than terminals in layer I of STG. The middle cortical layers are recipient of cortico-cortical and cortico-thalamic “feedforward” driving input, while layer I receives “feedback” modulatory pathways [e.g., (Hashikawa et al., 1995); reviewed in (Felleman and Van Essen, 1991; Jones, 1998; Abbott and Chance, 2005; Lee and Sherman, 2010)]. Large boutons that terminate in layer IV contain more synaptic vesicles and have a larger mitochondrial content than small boutons in layer I (Germuska et al., 2006), suggesting higher synaptic efficacy. The size of presynaptic terminals is correlated with the number of synaptic vesicles (Germuska et al., 2006; Zikopoulos and Barbas, 2007) and the probability of neurotransmitter release (Tong and Jahr, 1994; Murthy et al., 1997, 2001). One of the most efficient “drivers” of cortical neurons, especially in sensory areas, is the thalamic pathway that terminates in layer IV [(Rose and Metherate, 2005; Lee and Sherman, 2008; Cruikshank et al., 2010); reviewed in (Castro-Alamancos and Connors, 1997; Sherman and Guillery, 1998, 2002; Guillery and Sherman, 2002; Jones, 2002; Abbott and Chance, 2005; Silberberg et al., 2005; Lee and Sherman, 2010)]. Thus, as we have suggested previously (Germuska et al., 2006; Medalla et al., 2007) the laminar differences in size of axonal boutons from area 10 that terminate in auditory association cortex suggest differences in the strength of synaptic influence across cortical layers. This evidence suggests that area 10 can exercise diverse excitatory effects on STG association areas depending on the predominance of terminations in the upper or middle layers. In areas Ts1-2, the smaller upper layer terminals from area 10 suggest a predominant modulatory role of this specific pathway. Based on these synaptic relationships and findings of changes in the relative density of upper vs. deep layer terminals from area 10 to distinct STG areas, we have suggested that area 10 may drive activity in the temporal polar areas of STG through dense terminations in the middle-deep layers (Barbas et al., 2005).

Laminar terminations encounter specific microenvironments with regard to populations and dendritic segments of excitatory and inhibitory postsynaptic targets (reviewed in Peters, 1987; White, 1989; Callaway, 2002; Douglas and Martin, 2004). We found that most (about 80%) of the synapses in the pathway from area 10 to STG (areas Ts1-2) target spines (Germuska et al., 2006; Medalla et al., 2007), which are enriched on the dendrites of cortical excitatory neurons (Figures 5B, green; **5C,E,F** from Medalla et al., 2007). The laminar specificity of these spine-targeting boutons can influence which dendritic domains or population of neurons are innervated (reviewed in Silberberg et al., 2005; Spruston, 2008). Layer I is populated with the distal apical dendrites of neurons from the layers below, while the middle-deep layers consist mostly of proximal and basal dendrites of pyramidal neurons (Figure 5B, green P; Larkman and Mason, 1990; Larkman, 1991). Layer IV consists mostly of spiny stellate excitatory neurons (Figure 5B, s) that receive direct thalamic input in sensory cortices [(Peters et al., 1994); reviewed in (White, 1989)]. Thus, area 10 innervates mostly spines from apical dendrites of pyramidal neurons in layer I, but may interact with other dendritic segments and excitatory neuronal types in the deep layers.

### Synaptic interaction of area 10 with inhibitory neurons in STG

Inhibitory neurons in the primate cortex can be reliably identified and grouped by the expression of one of three calcium-binding proteins. One group of neurons expresses parvalbumin (PV, Figures 5A,B, red), a second group expresses calbindin (CB, Figures 5A,B, magenta), and a third group expresses calretinin (not shown). In primates, these neurochemical classes of inhibitory neurons represent distinct non-overlapping populations that differ in distribution, morphology, physiology and synaptic interactions with neighboring neurons (reviewed in Defelipe, 1997). Parvalbumin labels inhibitory neurons that innervate neighboring pyramidal neurons at their proximal dendrites or somata (basket cells) or the axon initial segments (chandelier cells) (Figures 5A,B, red; Defelipe et al., 1989b; Kawaguchi and Kubota, 1997; Thomson and Deuchars, 1997). Parvalbumin neurons have distinct fast-spiking firing properties and are the most reliably identified by physiologic methods [(Kawaguchi and Kubota, 1997; Krimer et al., 2005); reviewed in (Markram et al., 2004)]. The proximal innervation pattern and fast-firing properties of PV neurons suggest strong inhibition with rapid temporal dynamics for controlling the timing of spike output of pyramidal neurons (Rao et al., 1999; Constantinidis and Goldman-Rakic, 2002; Trevelyan and Watkinson, 2005). On the other hand, calbindin labels inhibitory neurons that innervate the distal dendrites and spines of excitatory neurons (Figures 5A,B, magenta; Defelipe et al., 1989a; Kawaguchi and Kubota, 1997; Peters and Sethares, 1997). CB inhibitory neurons are physiologically diverse but they are non-fast spiking and generally have slower firing dynamics than PV neurons (Kawaguchi and Kubota, 1997; Krimer et al., 2005; Zaitsev et al., 2005). It has been suggested that CB neurons engage a modulatory type of dendritic inhibition to selectively enhance the signal-to-noise ratio of relevant inputs within a cortical column (Wang et al., 2004). Interestingly, PV and CB inhibitory neurons in the primate cortex, including area 10 and the STG, have complementary laminar distributions (Hendry et al., 1989; Conde et al., 1994; Kondo et al., 1994; Gabbott and Bacon, 1996; Dombrowski et al., 2001; Medalla and Barbas, 2006). While PV neurons predominate in the middle-deep layers (IIIb–VI), CB neurons are densest in the upper layers (II–IIIa).

In addition to synapses on spines of presumed excitatory neurons, a smaller subset (~20% or less) of synapses from area 10 innervates dendrites of presumed inhibitory neurons in areas Ts1-2 (Figures 5D,G; Germuska et al., 2006; Medalla et al., 2007). By morphology, cortical inhibitory neurons have smooth or sparsely spiny dendrites [(Feldman and Peters, 1978; Kawaguchi et al., 2006); reviewed in (Peters et al., 1991; Fiala and Harris, 1999)], which are features that can readily be assessed at high-resolution, using three-dimensional electron microscopic methods (Figure 5G; Germuska et al., 2006; Medalla et al., 2007; Medalla and Barbas, 2009, 2010, 2012). We have found that area 10 innervates inhibitory neurons in layers I, II–IIIa, and IV of STG, with a trend for a slightly higher frequency in progressively deeper layers (II–IIIa and IV) compared to layer I (Germuska et al., 2006; Medalla et al., 2007). The middle-deep layers of STG are more densely populated by PV inhibitory neurons (Barbas et al., 2005). These layers are also innervated by excitatory “feedforward” cortico-cortical and cortico-thalamic fibers in the auditory cortex (Rose and Metherate, 2005; Lee and Sherman, 2008) and other cortical areas [(Melchitzky et al., 1999; Zhu and Connors, 1999; Beierlein et al., 2003; Gonchar and Burkhalter, 2003; Negyessy and Goldman-Rakic, 2005; Zikopoulos and Barbas, 2007; Cruikshank et al., 2010); reviewed in (White, 1989; Peters et al., 1994)]. We found that terminals from area 10 in layers II–IIIa of STG (areas Ts1-2) innervate CB neurons, as well as PV neurons (Figure 5B; Medalla et al., 2007). Thus, area 10 has the potential to engage two distinct modes of inhibition in STG: modulatory CB-mediated as well as rapid and strong PV-mediated inhibition.

In addition to inhibition at the site of termination in STG, inhibitory control via the area 10 pathway may also occur locally within area 10, by engaging inhibitory neurons that innervate projection neurons directed to STG (Figure 5A, pathway *d*). We have shown that projection neurons directed to STG areas arise mostly from the upper layers (II–III) of area 10 (Medalla et al., 2007). Pyramidal neurons in layers II–III extend their apical dendrites and arborize profusely in layer I [e.g., (Larkman and Mason, 1990; Larkman, 1991); reviewed in (Silberberg et al., 2005)]. Thus, the proximal and extensive distal apical domains of STG-directed projection neurons in area 10 are sites of potential synaptic innervation by the distinct classes of inhibitory neurons. In particular, the robust laminar overlap of STG projection neurons and CB neurons in layers II–IIIa suggests CB-mediated inhibition of auditory-directed projection neurons in area 10 (Figure 5A, magenta; Medalla et al., 2007).

In summary, pathway terminations from area 10 are diverse in distribution and synaptic features, which depend on the specific STG area and cortical layer of termination. These varied innervation patterns suggest that area 10 may have diverse influences on excitatory and inhibitory microcircuits in STG areas, allowing flexibility in prefrontal-auditory functional interactions for complex cognition.

## Functional implications of prefrontal-auditory pathways in high-order cognition

### Auditory connections of area 10 for complex cognition

The robust and diverse synaptic pathways from area 10 to the STG suggest a tight link between area 10 function and auditory processing. The evidence reviewed points to a specialized relationship of area 10 with the auditory association cortex as a key frontal “auditory field.” Area 10 receives information from almost all levels of processing in the STG—from the very detailed and early sensory processing in belt and parabelt areas to the complex high-order processing of acoustic stimuli for con-specific communication in temporal polar areas [e.g., (Poremba et al., 2004; Kusmierek and Rauschecker, 2009; Kikuchi et al., 2010); reviewed in (Romanski and Averbeck, 2009)]. The question thus arises as to how these connections are used in high-order cognitive functions mediated by area 10.

The frontal pole is thought to be part of the working memory network together with dorsolateral prefrontal areas 9/46, engaged for active maintenance of information for the task at hand (reviewed in Petrides, 2000; Barbas et al., 2002). Baddeley (1996) proposed two important components for working memory in humans—a visuospatial scratchpad and an articulatory loop. This is not surprising from an anatomical perspective, given that the working memory system in the lateral prefrontal cortex is indeed predominated posteriorly by visual-related periarcuate areas (caudal area 46 and FEF) and anteriorly by auditory-related areas (area 10 and mid-dorsolateral areas 46/9). Interestingly, this auditory-visual gradient along the rostro-caudal axis of lateral prefrontal areas is thought to coincide with a functional hierarchy by complexity of processing (reviewed in Petrides, 2000; Barbas et al., 2002; Burgess et al., 2007; Koechlin and Hyafil, 2007; Smith et al., 2007; Badre and D'Esposito, 2009). Area 10 is thought to be at the top of this hierarchy, which mediates the most complex and abstract cognitive tasks. This idea is exemplified in human functional neuroimaging studies that have shown specific recruitment of area 10 during complex multi-tasking, when one task must be temporarily suspended to attend to another task [(Koechlin et al., 1999; Burgess et al., 2000; Braver et al., 2003; Dreher et al., 2008; Tsujimoto et al., 2010); reviewed in (Burgess et al., 2007; Koechlin and Hyafil, 2007; Smith et al., 2007; Badre and D'Esposito, 2009)]. This can be illustrated for instance when a person interrupts cooking a meal to answer the phone and subsequently resumes cooking from where one left off. Moreover, the functional imaging studies have shown that multi-task functions and other related complex cognitive tasks mediated by area 10 rely on phonologic processing of “inner thoughts” for mental tracking of multiple information streams [e.g., (Bunge et al., 2005; Christoff et al., 2011); reviewed in (Burgess et al., 2007)]. Thus, area 10 may engage its strong auditory links for abstract representation of information in organized thought during complex cognitive tasks.

It is also interesting that the evolved complexity of cognition from monkeys to humans seems to parallel the cortical expansion of both the auditory system and the frontal pole. In particular, in humans the language cortices have evolved as specialized systems for verbal articulation as the frontal pole has also expanded laterally (Semendeferi et al., 2011; Sallet et al., 2013; Neubert et al., 2014). This evolutionary trend is also evident in the connections of area 10 in different species of non-human primates. Neural tracing in marmoset monkeys has shown a smaller representation of auditory connections in area 10 compared to macaque monkeys (Barbas et al., 1999; Burman et al., 2011). The above evidence is consistent with the idea that as the auditory system evolved in humans, area 10 kept pace with more extensive auditory connections. This trend is also reflected in behavior: as cognitive tasks in humans rely more on verbal information, more complex tasks can be tackled [e.g., (Frith, 1996; Wenzlaff and Wegner, 2000; Brewin and Smart, 2005; Bunge et al., 2005; Christoff et al., 2011); reviewed in (Knight et al., 1999; Allen et al., 2008; Winkler et al., 2009; Perrone-Bertolotti et al., 2014)]. By comparison with humans, monkeys have relatively poor capacity for working memory in the auditory domain (Ng et al., 2009; Scott et al., 2012, 2013a). Based on the functional and anatomical evidence, it is likely that the auditory interactions in a highly evolved area 10 are crucial to a role in high-order cognition in humans.

### Interaction of frontopolar and medial prefrontal “auditory fields” for cognitive control

In addition to area 10, auditory signals impinge on a wide spectrum of prefrontal areas (Figure 1). Particularly strong auditory connections are seen for medial prefrontal areas 32 and 25 in the ACC. Importantly, these prefrontal auditory “hotspots” are also robustly interconnected with each other through intrinsic prefrontal pathways (Barbas and Mesulam, 1985; Barbas and Pandya, 1989; Barbas et al., 1999; Medalla and Barbas, 2010). Thus, we have previously suggested that the prefrontal cortex may use auditory information either through direct connections with STG, or indirectly through local interconnections between auditory-related prefrontal areas (Barbas et al., 2005; Medalla et al., 2007; Medalla and Barbas, 2010).

Frontopolar area 10 and anterior cingulate areas, the rostral and medial frontal “auditory fields” that are most strongly interconnected with the STG, are also robustly linked with each other. In particular, a pathway from ACC area 32 innervates spines of excitatory neurons in area 10 through large and synaptically-effective boutons in layers II–III [(Medalla and Barbas, 2010); reviewed in (Barbas et al., 2013)]. These ACC boutons are larger than in the pathways linking area 10 with other dorsolateral prefrontal areas (46 or 9), and they are comparable in size to the “feedforward/driving” pathway terminations in layer IV of sensory cortices (Melchitzky et al., 2001; Anderson and Martin, 2002, 2005, 2006; Medalla et al., 2007), including the terminations from area 10 to layer IV of STG (Germuska et al., 2006). We have previously suggested that the large ACC terminations may drive and redirect activity in area 10 to help select relevant signals (presumably from auditory areas), and suppress noise for complex multi-task functions (Medalla and Barbas, 2010). This idea is consistent with the prominent role of the ACC in allocating attention and in task-switching, especially when cognitive demand is high (reviewed in Barbas and Zikopoulos, 2007; Botvinick, 2007; Lee et al., 2007; Schall and Boucher, 2007). Interestingly, the ACC has a demonstrated influence in several high-order auditory-related functions by affecting activity in auditory cortices. Microstimulation of ACC evokes species-specific vocalization in monkeys and affects activity in auditory cortices [(Muller-Preuss et al., 1980; Muller-Preuss and Ploog, 1981); reviewed in (Vogt and Barbas, 1988)]. In humans, correlated gamma-band activity in ACC and auditory areas suggests functional coupling between these cortices during demanding cognitive tasks (Mulert et al., 2007).

The ACC-frontopolar-auditory network may mediate high-order filtering of auditory processing to allow communication in a noisy environment. Such filtering has been discussed for the auditory modality, in general (reviewed in Conway et al., 2001; Denham and Winkler, 2006; Jaaskelainen et al., 2007; Micheyl et al., 2007). The pathways that link area 10 with the auditory areas may help keep track of internal thoughts, which is important for working memory and problem solving [e.g., (Brewin and Smart, 2005); reviewed in (Wenzlaff and Wegner, 2000)]. This hypothesis is consistent with findings that the ACC and area 10 are activated during mental tracking of multiple tasks (reviewed in Burgess et al., 2007). The anatomical evidence supports this hypothesis, but behavioral and functional studies that employ auditory-related tasks are needed to investigate the role of auditory input, and the specialized projections from ACC, for cognitive processing in area 10.

### Prefrontal-auditory pathway disruption in disease

Pathology in the prefrontal-auditory network has been implicated in schizophrenia, a disease characterized by high distractibility, disordered thought patterns and auditory hallucinations (reviewed in Cohen et al., 1996; Honey and Fletcher, 2006; Allen et al., 2008). *Post-mortem* studies in brains of schizophrenic patients show that specific markers for populations of inhibitory and excitatory neurons are diminished in auditory-related ACC and mid-dorsolateral prefrontal areas, disrupting the excitatory and inhibitory balance necessary for normal cognitive functions (reviewed in Benes, 2000; Beasley et al., 2002; Volk and Lewis, 2002; Vogels and Abbott, 2007; Fornito et al., 2009; Eisenberg and Berman, 2010). For instance, the ACC shows a decrease in pyramidal neuron density in the deep layers (Benes et al., 2001) and reduced overall activity in schizophrenia (Fletcher et al., 1999; Kerns et al., 2005; Snitz et al., 2005; Allen et al., 2007; Leicht et al., 2010). The deep layers of ACC give rise to projections to lateral prefrontal cortices in monkeys, in a pattern expected to hold for humans based on the predictions of the structural model for connections (Barbas and Rempel-Clower, 1997). Based on available data on the synaptic circuits within the prefrontal-auditory network, we have speculated that pathologic hypofunction in ACC may weaken its output to inhibitory neurons in other auditory-related prefrontal cortices such as frontopolar area 10 and mid-dorsolateral areas 9/46 (Medalla and Barbas, 2009, 2010). The strong influence of the ACC on CB inhibitory neurons, in particular, suggests a mechanism to suppress noise (Wang et al., 2004). By the same principle, hypofunction especially in the deep layers of ACC may reduce excitation to frontopolar area 10, which is engaged when keeping track of internal thoughts to perform multiple tasks in humans (reviewed in Burgess et al., 2007; Koechlin and Hyafil, 2007; Smith et al., 2007; Badre and D'Esposito, 2009). Weakening of these interactions may account for the high distractibility and disordered thought patterns in schizophrenia (reviewed in Cohen et al., 1996; Honey and Fletcher, 2006; Allen et al., 2008). Finally, the relative activation of ACC and auditory cortices appears to help distinguish actual from inner speech in humans, in functions that are disrupted in schizophrenic patients who experience auditory hallucinations (Frith et al., 1995; McGuire et al., 1995). The strong and specific anatomic pathways interlinking prefrontal and auditory cortices reviewed here thus suggest a key role of these interactions in high-order cognition, and may help explain the impairments in processing of “inner thoughts” that account for the distractibility, disordered thought process and auditory hallucinations in schizophrenia.

### Conflict of interest statement

The authors declare that the research was conducted in the absence of any commercial or financial relationships that could be construed as a potential conflict of interest.
